# Equilibrium and Kinetic Studies of Cd^2+^ Biosorption by the Brown Algae *Sargassum fusiforme*

**DOI:** 10.1371/journal.pone.0095242

**Published:** 2014-04-15

**Authors:** Hui-Xi Zou, Nan Li, Li-Hua Wang, Ping Yu, Xiu-Feng Yan

**Affiliations:** Zhejiang Provincial Key Lab for Subtropical Water Environment and Marine Biological Resources Protection, College of Life and Environmental Science, Wenzhou University, Wenzhou, China; University of New South Wales, Australia

## Abstract

A fundamental investigation of the biosorption of Cd^2+^ from aqueous solution by the edible seaweed *Sargassum fusiforme* was performed under batch conditions. The influences of experimental parameters, such as the initial pH, sorption time, temperature, and initial Cd^2+^ concentration, on Cd^2+^ uptake by *S. fusiforme* were evaluated. The results indicated that the biosorption of Cd^2+^ depended on the initial Cd^2+^ concentration, as well as the pH. The uptake of Cd^2+^ could be described by the Langmuir isotherm model, and both the Langmuir biosorption equilibrium constant and the maximum biosorption capacity of the monolayer decreased with increasing temperature, thereby confirming the exothermic character of the sorption process. The biosorption kinetics follows the pseudo-second-order kinetic model, and intraparticle diffusion is the sole rate-limiting step for the entire biosorption period. These fundamental equilibrium and kinetic results can support further studies to the removal of cadmium from *S. fusiforme* harvested from cadmium-polluted waters.

## Introduction

Cadmium is one of the most toxic metals that affects the environment and human beings. Mining and metallurgy of cadmium, cadmium electroplating processes, battery and accumulator manufacturing, and pigments and ceramics industries produce wastewater that contains undesirable amounts of Cd^2+^ ions [1]. Moreover, cadmium can accumulate in the tissue of plants growing in industrially polluted areas, and this metal can be transferred to humans through food chains [2].

The brown seaweed *Sargassum fusiforme* (Sargassaceae, Phaephyceae) is among the most mineral-rich algae that is high in calcium, iodine, and magnesium, and is commonly found along the coastlines of China, Japan, and Korea [3]. In ancient Asia, this seaweed was not only used as a traditional medicine but was also enjoyed as a popular food for its nutty flavor. This alga, in great demand, is also cultivated in these counties, especially in China, where the cultivation area was 2.6% (2,482 ha) of the entire coastal area for the commercial cultivation of seaweeds with a total production of 32,000 tonnes (freshweight) [4].

Huerta-Díaz *et al.* [5] determined that *Sargassum* can accumulate large amounts of divalent metals. In this process of biosorption, the cell wall plays an important role in metal binding because it contains high concentrations of polysaccharides [6], including alginate, which is the main polysaccharide responsible for the biosorption of metals in *Sargassum* [7], [8].

The biosorption of cadmium ions by different living and dead types of algae has been extensively studied [9]–[14]. Numerous reports have recently suggested approaches that use living and nonliving algae for accumulating or removing heavy metals from aqueous solutions. However, neither the equilibrium nor the kinetic modeling of Cd^2+^ biosorption by dried *S. fusiforme* has been investigated.

Thus, in this work, we studied the biosorption of cadmium ions by *S. fusiforme* by investigating the influences of different experimental parameters on cadmium uptake, including the initial Cd^2+^ concentration, the initial pH, the sorption time, and the temperature. The experimental data were correlated with different kinetic and biosorption models, and the corresponding parameters and their temperature dependence were determined. These parameters provide fundamental information for use in further studies to remove cadmium ions from *S. fusiforme* harvested from cadmium-polluted waters.

## Materials and Methods

### Preparation of *S. fusiforme*

*S. fusiforme* samples were collected from the Northeastern coast of Wenzhou, China (28.0°N, 121.2°E) in October 2012. This location is not privately-owned or protected in any way, thus no specific permissions were required, and the field studies did not involve endangered or protected species. This sample was washed extensively with distilled water to remove particulate material from its surface, oven-dried at 333 K for 24 h, placed in desiccators, and allowed to cool to room temperature.

To remove divalent ions present in this native *S. fusiforme* sample and replace them with sodium ions (which can be easily displaced by metal ions) [5], [6], 50 g of dried *S. fusiforme* sample was treated with 0.1 mol L^−1^ HCl (500 mL) and then 0.1 mol L^−1^ NaOH (500 mL) for 1 h under slow stirring. The sample was then washed several times with deionized water until the pH of the rinse water was less than 10 [15]; this *S. fusiforme* sample was dried again at 333 K for 24 h.

Standard sampling techniques were applied to ensure the homogeneity of samples used in these experiments. The dried sample was chopped and sieved, and the 0.5–0.6 mm fraction was selected for use in the sorption tests.

### Chemicals

All solutions were prepared from deionized water and an analytical grade salt of Cd(NO_3_)_2_·4H_2_O (Shanghai Aladdin Co., China). The solution pH was measured with a pH meter (METTLER TOLEDO, model Delta 320, China) and was adjusted to 5 with 0.1 mol L^−1^ HCl and/or 0.1 mol L^−1^ NaOH.

### Biosorption experiments

Batch biosorption experiments were performed in 250 mL stoppered conical flasks that contained 0.500 g of *S. fusiforme* and 100 mL of Cd^2+^ standard solution. These test solutions were agitated in a 120 rpm controlled-temperature reciprocating shaker reciprocating shaker (SHA-B, China) at a constant temperature for 2 h. The influence of the initial solution pH (1–8), the contact time (2–120 min), and the initial Cd^2+^ concentration (30–300 mg L^−1^) were investigated. We conducted biosorption equilibrium experiments by combining 0.500 g *S. fusiforme* with 100 mL Cd^2+^ standard solution at different initial concentrations and at various temperatures (278 K, 288 K, 298 K, and 308 K). Kinetic studies were performed by mixing 0.500 g *S. fusiforme* with 300 mg L^−1^ Cd^2+^ solution in a series of 250 mL stoppered conical flasks. These flasks were removed at certain time intervals as shown in figures. After biosorption, the *S. fusiforme* was separated by centrifugation; the supernatant fluids were analyzed and the residual Cd^2+^ concentrations were measured using an atomic absorption spectrophotometer (Persee Tas 986, China) with a detection limit of 1.0 ppm at a wavelength of 228.8 nm. All samples were detected in duplicate. The accuracies for the Cd^2+^ analyses were achieved by comparing the measured concentrations to the added Cd^2+^ standards. The precisions and accuracies of the determination were estimated by replicating analysis (n = 5) of QC samples at three concentrations levels.

The amount of Cd^2+^ adsorbed at equilibrium, *q*_e_ (mg g^−1^), i.e., the amount of Cd^2+^ adsorbed per unit mass of *S. fusiforme* at equilibrium (mg g^−1^), was calculated by:
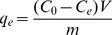
(1)where *C*_0_ is the initial concentration of Cd^2+^ (mg L^−1^), *C*_e_ is the equilibrium concentration of Cd^2+^ in solution (mg L^−1^), *V* is the volume of the solution (mL), and *m* is the mass of dry algae (g). Preliminary experiments had shown that cadmium biosorption losses on the flask walls and the filter paper were negligible.

### Data analyses

Equilibrium biosorption isotherms provide fundamental information related to the design of biosorption systems. An isotherm indicates how adsorbate molecules are distributed between the liquid phase and the solid phase when the biosorption process reaches equilibrium [16]. In the present investigation, the biosorption data were analyzed using Langmuir and Freundlich isotherm models. The Langmuir isotherm model is based on the assumption that a monolayer adsorbs onto a homogeneous surface containing a finite number of biosorption sites with uniform strategies of biosorption and no transmigration of adsorbate on the plane of the surface [17]. The linear form of the Langmuir isotherm model is given by the following equation:
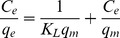
(2)where *C*_e_ is the equilibrium concentration of Cd^2+^ in solution (mg L^−1^), *q*_m_ is the maximum biosorption capacity of the monolayer (mg g^−1^), and *K*_L_ is the Langmuir biosorption equilibrium constant related to the energy of biosorption (L mg^−1^). The values of *q*_m_ and *K*_L_ can be evaluated from a linear plot of *C*_e_/*q*_e_ as a function of *C*_e_.

The Freundlich isotherm model is an empirical equation used to describe heterogeneous systems [18], and it is represented by the following linear equation: 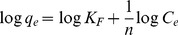
(3)where *K*_F_ ((mg g^−1^) (mg^−1^ L)^1/n^) and 1/*n* are Freundlich constants related to adsorbent biosorption capacity and biosorption intensity, respectively. The *K*_F_ and *n* values can be calculated from the intercept and the slope of a liner plot of log *q*_e_ as a function of log *C*_e_.

To analyze the biosorption kinetics of Cd^2+^, pseudo-first-order and pseudo-second-order kinetics models were applied in this study. The equation of the pseudo-first-order kinetics model can be written as:
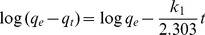
(4)where *q*_e_ and *q*_t_ are the amount of Cd^2+^ adsorbed (mg g^−1^) at equilibrium and at time *t*, *k*_1_ is the pseudo-first-order equilibrium rate constant (min^−1^), and *t* is the contact time (min). However, to adjust Eq. (4) to fit the experimental data, the value of *q*_e_ must be pre-estimated through extrapolation of the experimental data to t ∝ ∞.

The pseudo-second-order kinetic rate equation is expressed as:
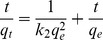
(5)where *k*_2_ is the pseudo-second-order rate constant (g mg^−1^ min^−1^). A plot of *t/q_t_* versus *t* should be a straight line if pseudo-second-order kinetics are applicable, and *q*_e_ and *k*_2_ can be determined from the slope and intercept of this plot, respectively.

Because the pseudo-first-order and pseudo-second-order kinetics models cannot identify the diffusion mechanism, the kinetic biosorption data were further evaluated using an intraparticle diffusion model. The intraparticle diffusion model, as expressed by Weber and Morris [19], can be described as:
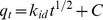
(6)where *k*_id_ is the intraparticle diffusion rate constant (mg g^−1^ min^−1/2^) and *C* is the intercept.

## Results and Discussion

### Precisions and recoveries of the Cd^2+^ analyses

The linear regression result of absorbance against Cd^2+^ concentration detected by atomic adsorption spectrophotometer is 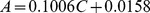, the correlation coefficient is 0.9997, where *A* is the absorbance of Cd^2+^, *C* is the concentration of Cd^2+^ (the linear range of Cd^2+^ is from 1 mg L^−1^ to 8 mg L^−1^). The precision and accuracy results are summarized in Table 1. The precision of the determination is described as relative standard deviation (RSD) among each assay. The accuracy is evaluated by the recovery values, which described as a percentage error of the measured concentrations vs. QC added concentrations. As can be seen from Table 1, the RSD values are lower than 0.6%, and the recovery values are among the range of 96.3–97.1%. So the determination shows desired precision and accuracy.

**Table 1:** Precision and accuracy data for the Cd^2+^ analyses.

QC added (mg L^−1^)	Mean concentration (mg L^−1^)	RSD (%)	Recovery (%)
30	28.9	0.2	96.3
150	145.7	0.3	97.1
300	289.3	0.6	96.4

### Effect of initial pH

**Figure 1:**
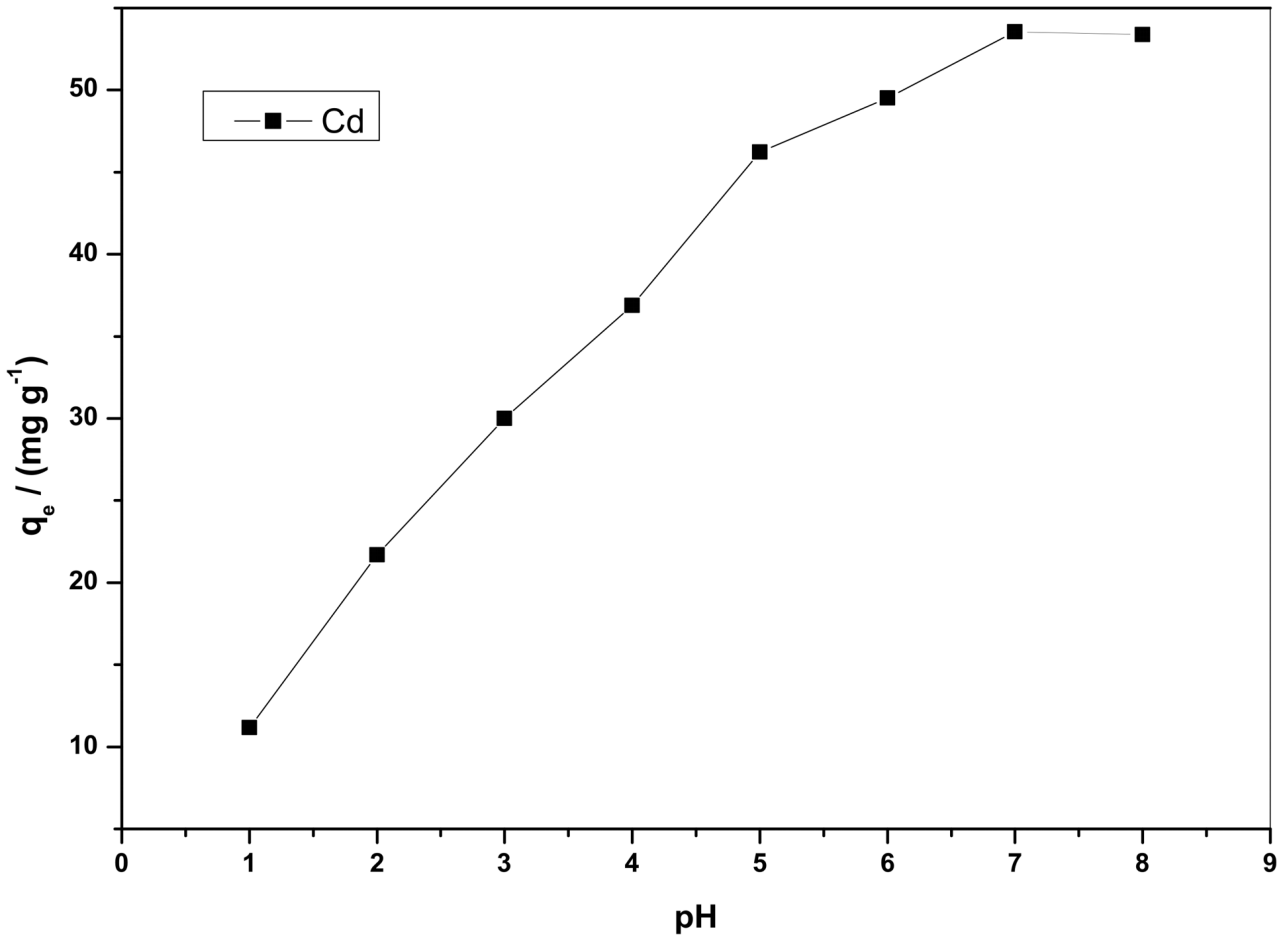
Effect of initial pH on the biosorption of Cd^2+^ by *Sargassum fusiforme*.

The initial pH is one of the most important parameters controlling the biosorption of metal ions because it affects both the surface binding sites of the adsorbent and the ionization process of the metal ions [12]. In the present biosorption system, the effect of initial pH was evaluated within the range of 1.0–8.0 at a concentration of 300 mg L^−1^ of Cd^2+^. As shown in Fig. 1, the increase of Cd^2+^ biosorption efficiency with pH is made until to the optimum pH value is reached (pH 7). Solution pH is known to influence cell surface metal binding sites and metal chemistry in water. Hence, the biosorption of Cd^2+^ onto *S. fusiforme* is influenced primarily by the surface charge on the cell wall of the brown algae, which is influenced by the solution pH. At low pH levels, functional groups from cell walls were associated closely with the hydronium ions of H_3_O^+^ and restricted the approach of metal cations as a result of repulsive forces [20]. As the pH increased, more functional groups such as carboxyl, phosphate, imidazole and amino groups in the cell wall would be exposed, and their negative charges would subsequently attract metallic ions with positive charges that biosorb onto the cell surface [21]. At higher pH values (7.0–8.0), the surface of *S. fusiforme* may reach the zero-point charge, or isoelectric point, and thus not affect the Cd^2+^ biosorption [22]. So pH = 7 is the optimum value.

### Effect of contact time

**Figure 2:**
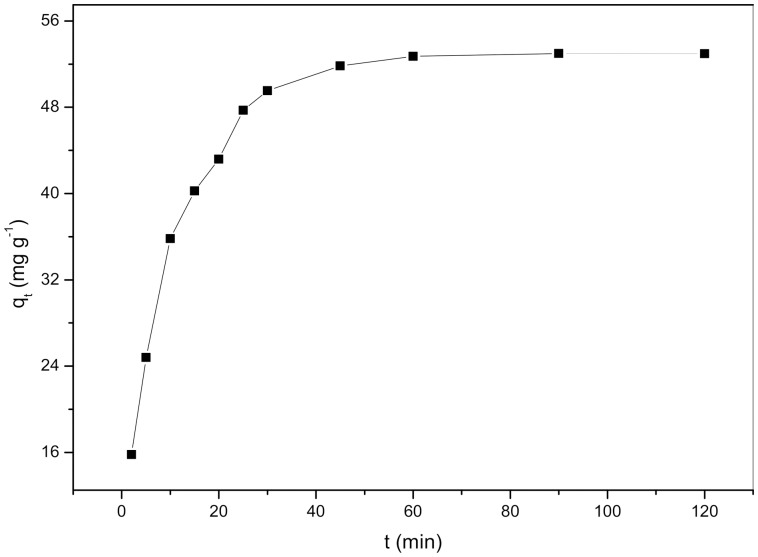
Effect of contact time on the biosorption of Cd^2+^ by *Sargassum fusiforme*.

The effect of contact time on the biosorption of Cd^2+^ by *S. fusiforme* was investigated using a constant initial Cd^2+^ concentration of 300 mg L^−1^ at 298 K. As shown in Fig. 2, the biosorption of Cd^2+^ increases sharply during the first 25 min, increases more slowly up to 60 min, and then remains almost constant after 60 min. The initial rapid stages are attributed to the abundant availability of active binding sites on the cell wall of the *S. fusiforme*, and, with gradual occupancy of these sites, the biosorption becomes less efficient in the later stages [23]. Although the equilibrium time was 60 min for all the biosorbents used in this study, the contact time was fixed at 120 min for the remainder of the batch experiments to ensure that equilibrium was reached.

### Effect of initial Cd^2+^ concentration

**Figure 3:**
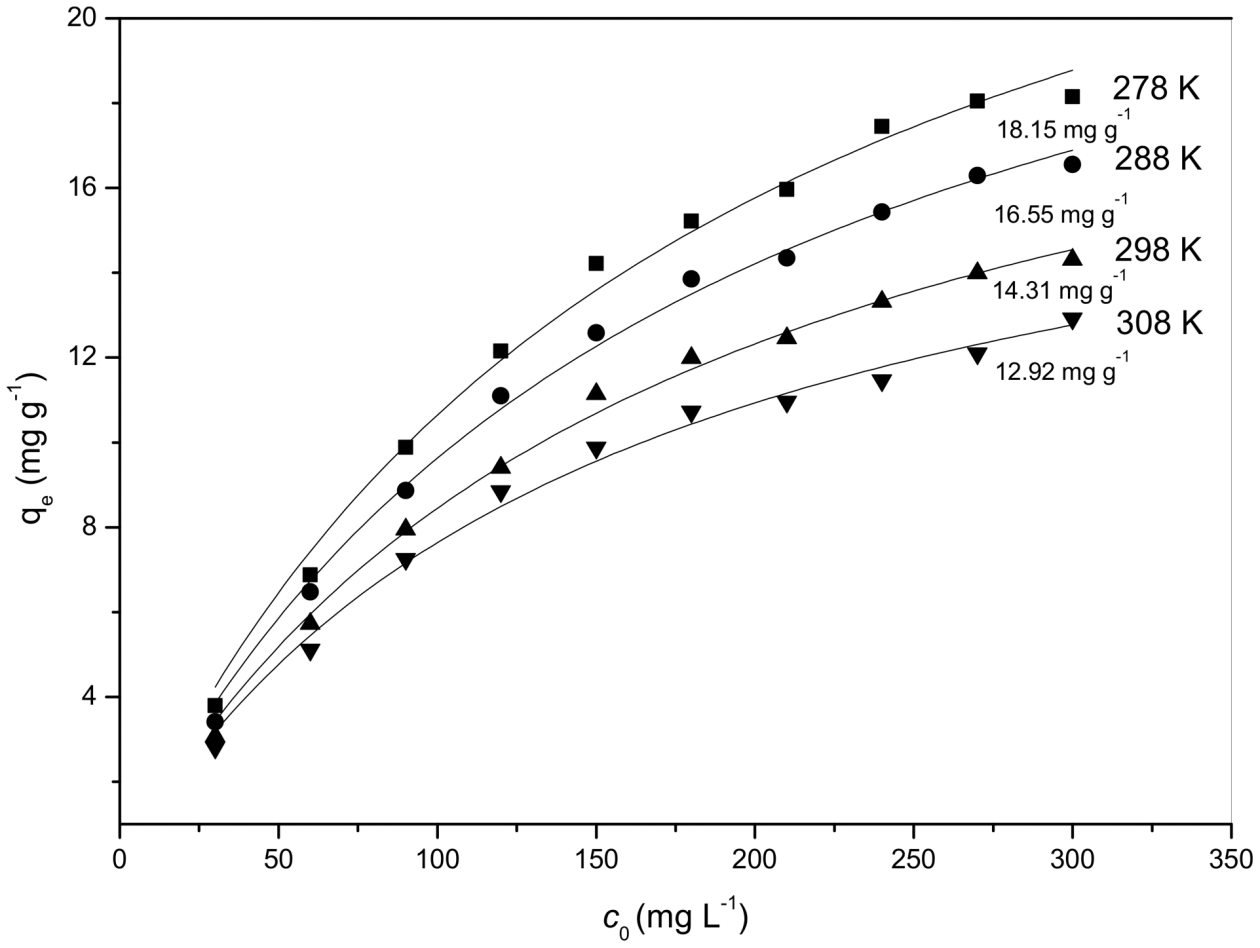
Effect of initial Cd^2+^ concentration on the biosorption of Cd^2+^ by *Sargassum fusiforme*.

Figure 3 shows the effect of the initial Cd^2+^ concentration on the biosorption of Cd^2+^ by *S. fusiforme* and indicates that the biosorption capacities of *S. fusiforme* increased with the initial Cd^2+^ concentration. When the initial Cd^2+^ concentration was increased from 30 to 300 mg L^−1^ (298 K), the biosorption capacity of *S. fusiforme* increased from 16.2 to 33.1 mg g^−1^. Higher initial Cd^2+^ concentrations not only provide a larger driving force to overcome all mass transfer resistances of Cd^2+^ between the aqueous and solid phases but also result in a higher probability of collision between Cd^2+^ and binding sites from *S. fusiforme* surfaces [24].

Additionally, also in Fig. 3, the amount of Cd^2+^ adsorbed by *S. fusiforme* at equilibrium (*q*_e_) is decrease with the increase of the temperature. For example, at the initial Cd^2+^ concentration of 300 mg L^−1^, the value of *q*_e_ is decreased from 18.15 mg g^−1^ to 12.92 mg g^−1^, which means that the process of Cd^2+^ sorption by *Sargassum* sp. is exothermic, as observed for other systems such as Cd^2+^-*Sargassum* sp. [24], Cd^2+^-*Sargassum baccularia* [25], and Cd^2+^-*Sargassum fluitans* [26]. Therefore, an increase in temperature is beneficial for removing Cd^2+^ from *S. fusiforme*.

### Equilibrium study

**Figure 4:**
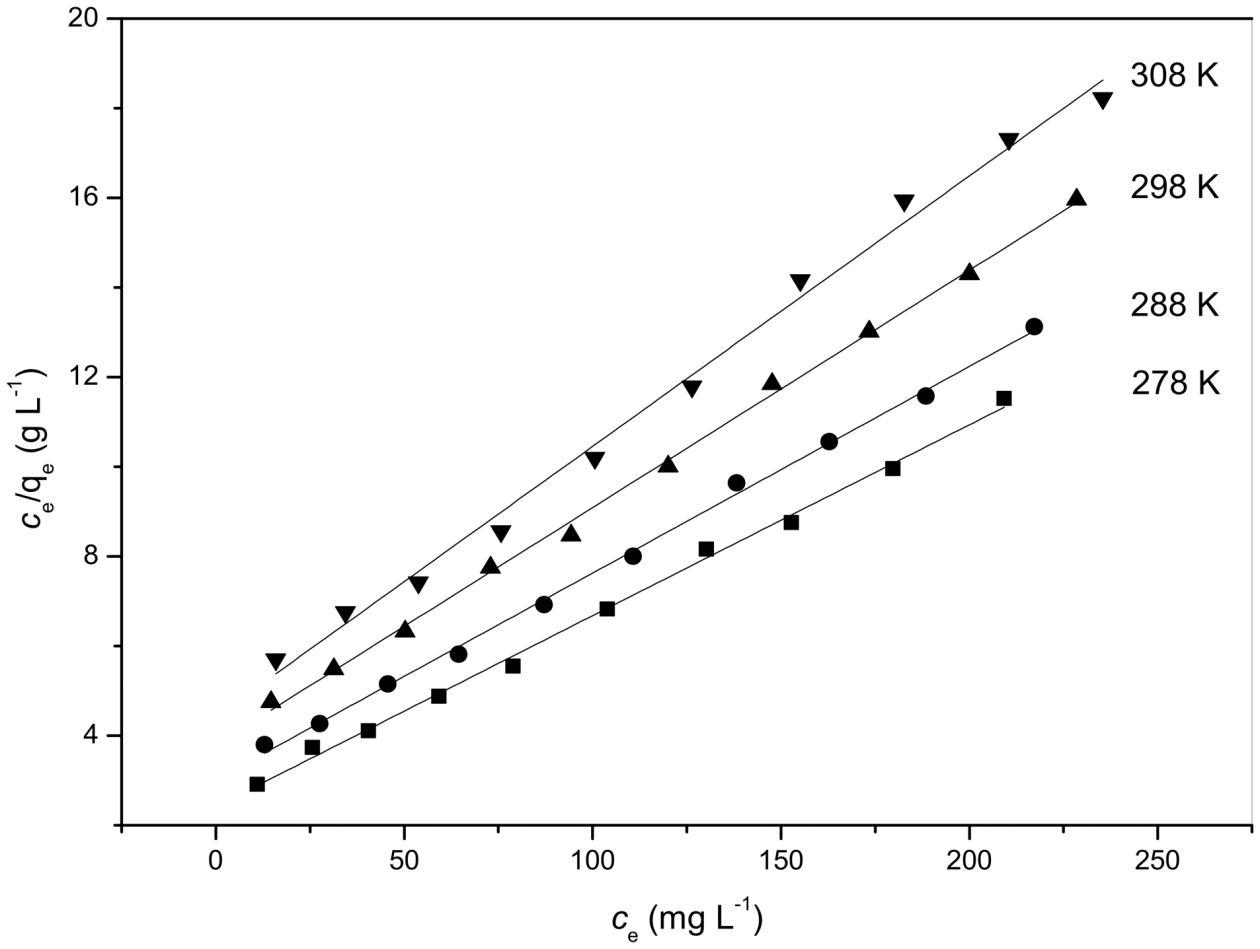
Langmuir isotherm plots for Cd^2+^ biosorption at different temperatures.

**Figure 5:**
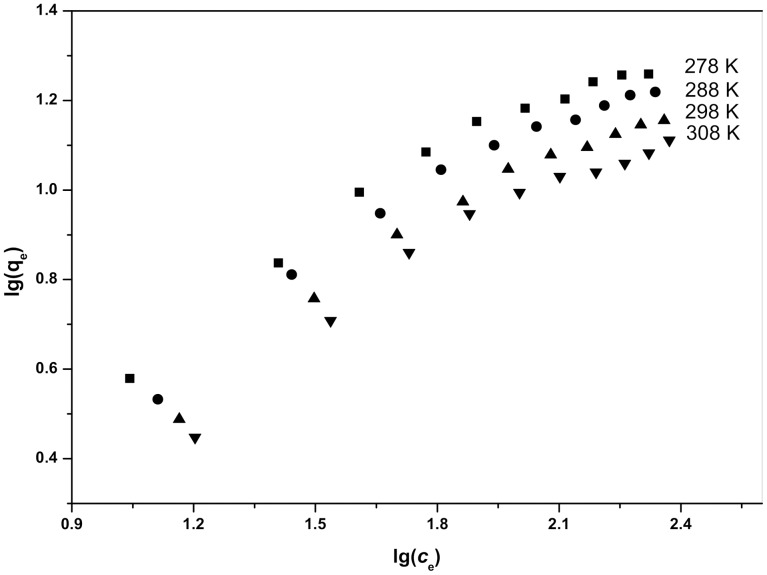
Freundlich isotherm plots for Cd^2+^ biosorption at different temperatures.

The Langmuir isotherm plots and Freundlich isotherm plots for Cd^2+^ biosorption at different temperatures are shown in Fig. 4 and Fig. 5, their values are also listed in Table 2. As shown in Table 2, the values of *n* are all within the range of 1–10, further indicating that these biosorption processes are favorable under the previously described conditions [27].

**Table 2:** Isotherm parameters for the biosorption of Cd^2+^ in solution at different temperatures.

	Langmuir			Freundlich		
Temperature (K)	*q*_m_ (mg·g^−1^)	*K*_L_ (L·mg^−1^)	*R* ^2^	*K*_F_ (mg·g^−1^)·(mg^−1^·L)^1/n^	*n*	*R* ^2^
278	23.481±2.03	0.0176±0.014	0.9966	3.371±0.035	1.895±0.055	0.9530
288	21.685±1.99	0.0153±0.009	0.9978	1.023±0.022	1.850±0.035	0.9525
298	18.883±1.58	0.0140±0.006	0.9979	0.851±0.019	1.849±0.023	0.9556
308	16.571±3.17	0.0137±0.021	0.9931	0.754±0.095	1.865±0.055	0.9443

In the present study, the correlation coefficient (R^2^) was used to confirm the best-fit isotherm for this biosorption system. The results are shown in Table 2. The Langmuir isotherm model appears to fit the isotherm data better than the Freundlich model because the correlation coefficients are higher for Langmuir's model. The fit of equilibrium data to the Langmuir model may indicate that the biosorption of Cd^2+^ onto *S. fusiforme* is monolayer biosorption. As also shown in Table 2, the values of the Langmuir constants *q*_m_ and *K*_L_ all decreased with increasing temperature, which suggests that both the biosorption capacity and the energy of biosorption tend to decrease with increasing temperature, thereby confirming the exothermic character of the sorption process.

Because *K*_L_ is an equilibrium constant, its dependence on temperature can be used to estimate both the enthalpy change (Δ*H*) and the entropy change (Δ*S*) associated with the biosorption process.
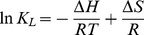
(7)


The plot of ln *K*_L_ as a function of 1/*T* yielded a straight line from which a Δ*H* value equal to −8.1 kJ mol^−1^ and a Δ*S* value equal to −2.69 J mol^−1^ K^−1^ were calculated.

A comparison of the values of Δ*H* and Δ*S* shows that the negative enthalpy change compensates for a sufficient amount of the entropy loss that the Gibbs energy changes are less than zero. Therefore, the biosorption of Cd^2+^ by *S. fusiforme* is a spontaneous and enthalpically driven process.

### Biosorption kinetics

**Figure 6:**
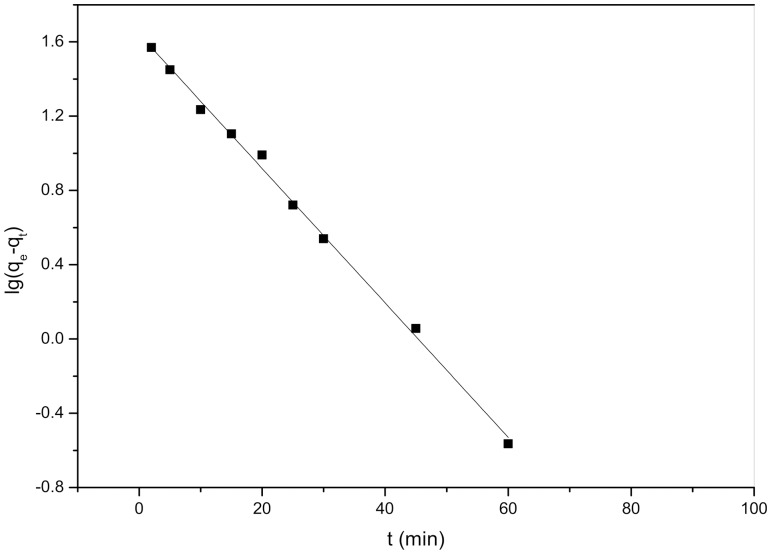
Plots of pseudo-first-order kinetic model equation for the biosorption of Cd^2+^ on the *Sargassum fusiforme*.

**Figure 7:**
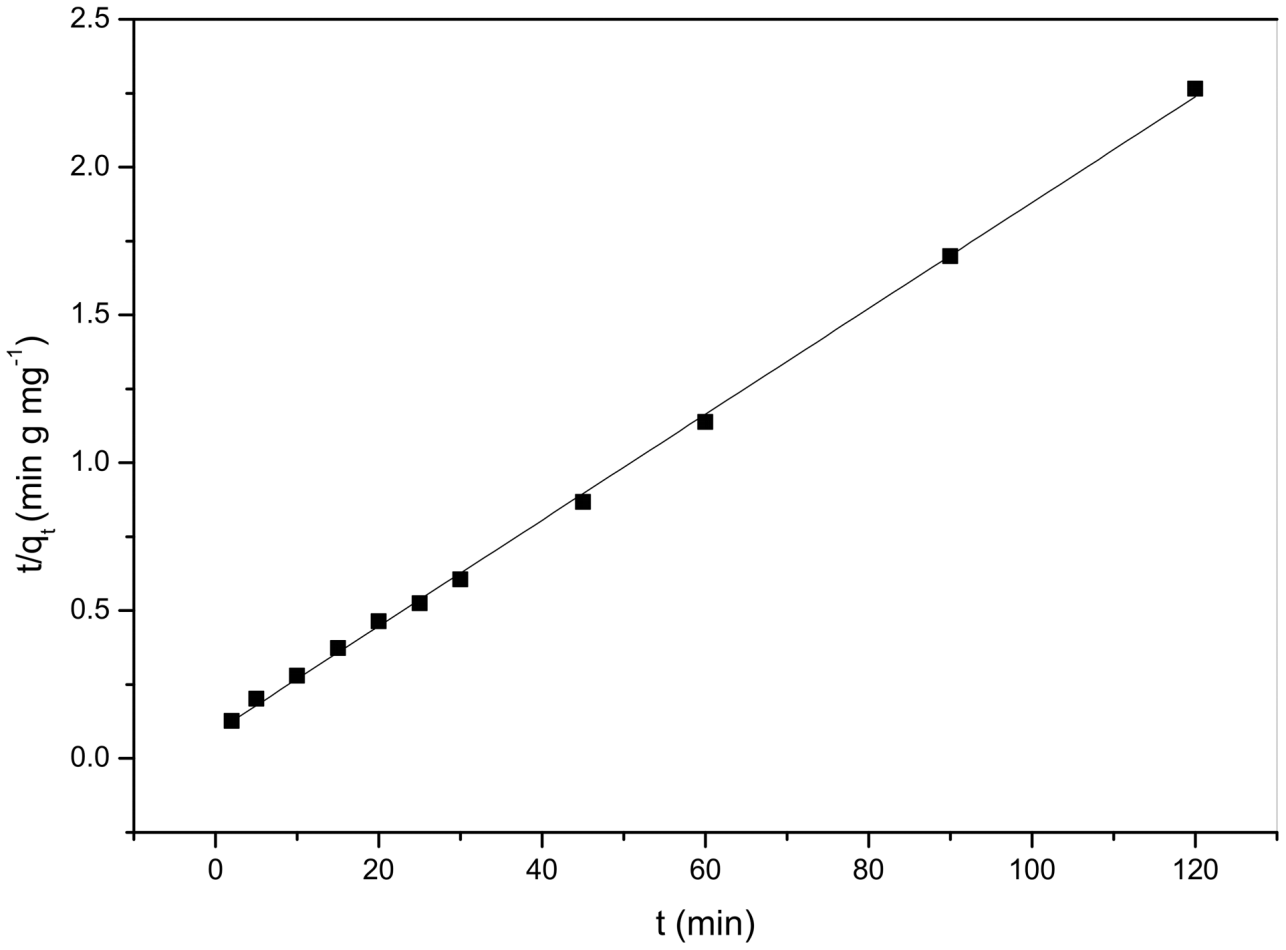
Plots of pseudo-second-order kinetic model equation for the biosorption of Cd^2+^ on the *Sargassum fusiforme*.

Figure 6 and Fig. 7 present pseudo-first-order and pseudo-second-order kinetics plots for the biosorption of Cd^2+^ on *S. fusiforme*, respectively. The kinetics parameters determined by the two models and the corresponding correlation coefficients (*R* ^2^) are listed in Table 3. In this study, on the basis of the higher *R* ^2^ values, the pseudo-second-order kinetic model gives a better fit to the biosorption data than does the pseudo-first-order model. This result demonstrates that the rate-limiting step for biosorption may be chemical biosorption involving covalent forces through sharing or the exchange of electrons between *S. fusiforme* and Cd^2+^ metal ions. On the basis of physical biosorption capacity, the pseudo-first-order rate equation has been widely used to describe the biosorption of organic pollutants from wastewater [28]. However, the pseudo-second-order rate equation is based on chemical biosorption, especially chemical bonding among divalent metal ions and polar functional groups such as aldehydes, ketones, and acids on biomass [29]. In this study, algal acidic functional group is assumed to be responsible for the cation-biosorption capacity of *S. fusiforme*.

**Table 3:** Parameters of pseudo-first-order and pseudo-second-order kinetic models.

Model	Regression equation	equilibrium rate constant	*R* ^2^
Pseudo-first-order	Y = −0.036X+1.64	*k*_1_ (min^−1^) = 0.083	0.9763
Pseudo-second-order	Y = 0.018X+0.090	*k*_2_ (g·mg^−1^·min^−1^) = 0.0040	0.9991

Biosorption always occurs through three consecutive steps [30]: (1) diffusion across the liquid film surrounding the adsorbent particles, i.e., external diffusion of Cd^2+^ toward *S. fusiforme*; (2) diffusion in the liquid contained in the pores and/or along the pore walls, which is so-called internal diffusion or intraparticle diffusion, i.e., the diffusion of Cd^2+^ at the rough cell surface of *S. fusiforme*; and (3) biosorption and desorption between the adsorbate and active sites, which includes a number of passive accumulation processes and may include biosorption, ion exchange, coordination, complexation, chelation, and microprecipitation.

According to the theory behind the intraparticle diffusion model, a plot of *q*_t_ against *t* ^1/2^ should yield a straight line if intraparticle diffusion is involved in the biosorption process; furthermore, if the straight line passes through the origin, then intraparticle diffusion is the sole rate-controlling step. Otherwise, the biosorption process may involve some other mechanisms in addition to intraparticle diffusion [31].

**Figure 8:**
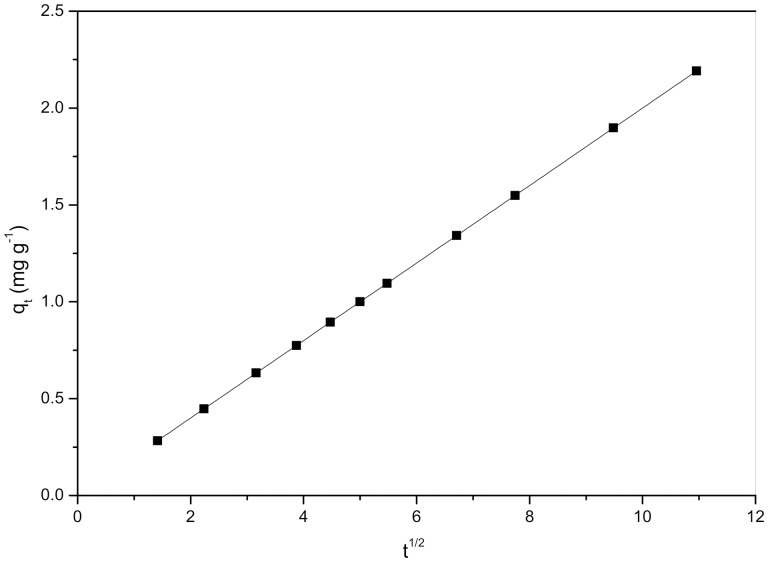
Plots of intra-particle diffusion kinetic model equation for the biosorption of Cd^2+^ on the *Sargassum fusiforme*.

As shown in Fig. 8, the linear regression equation is Y = 0.2X−2.7×10^−7^, the coefficient for the intra-particle diffusion model is equal to 1, the small value of the intercept could be negligible, and thus intraparticle diffusion is the sole rate-limiting step.

## Conclusions

In the present study, the biosorption of Cd^2+^ onto *S. fusiforme* could be described by the Langmuir biosorption model. Both the Langmuir biosorption equilibrium constant and the maximum biosorption capacity of the monolayer decreased with increasing temperature, thereby confirming the exothermic characteristic of the sorption process. In addition, the biosorption of Cd^2+^ by *S. fusiforme* is a spontaneous and enthalpically driven process.

The biosorption kinetics of Cd^2+^ onto *S. fusiforme* followed a pseudo-second-order kinetic model, which was based on chemical biosorption. Because the regression of the intra-particle diffusion model was fine, the diffusion rate is controlled solely by the intraparticle diffusion process.
